# Acceptability of Four Intervention Components Supporting Medication Adherence in Women with Breast Cancer: a Process Evaluation of a Fractional Factorial Pilot Optimization Trial

**DOI:** 10.1007/s11121-024-01711-9

**Published:** 2024-07-26

**Authors:** Sophie M. C. Green, Nikki Rousseau, Louise H. Hall, David P. French, Christopher D. Graham, Kelly E. Lloyd, Michelle Collinson, Pei Loo Ow, Christopher Taylor, Daniel Howdon, Robbie Foy, Rebecca Walwyn, Jane Clark, Catherine Parbutt, Jo Waller, Jacqueline Buxton, Sally J. L. Moore, Galina Velikova, Amanda Farrin, Samuel G. Smith

**Affiliations:** 1 Leeds Institute of Health Sciences, University of Leeds, Leeds, UK LS2 9NL; 2 Leeds Institute of Clinical Trials Research, University of Leeds, Leeds, UK LS2 9NL; 3 Manchester Centre for Health Psychology, University of Manchester, Manchester, UK M13 9PL; 4 Department of Psychological Sciences & Health, University of Strathclyde, Glasgow, G1 1QE Scotland; 5 Department of Clinical and Health Psychology, Leeds Teaching Hospitals NHS Trust, Leeds, LS9 7TF UK; 6 Medicines Management and Pharmacy Services, Leeds Teaching Hospitals NHS Trust, Leeds, LS9 7TF UK; 7 Wolfson Institute of Population Health, Queen Mary University of London, London, UK; 8 Independent, Leeds, UK; 9 Leeds Institute of Medical Research at St James’s, University of Leeds, St James’s University Hospital, Leeds, UK LS9 7TF; 10 Leeds Cancer Centre, Leeds Teaching Hospitals NHS Trust, St James’s University Hospital, Leeds, UK LS9 7TF

**Keywords:** Process evaluation, Acceptability, Factorial, Breast cancer, Medication adherence

## Abstract

Adjuvant endocrine therapy (AET) reduces mortality in early-stage breast cancer, but adherence is low. We developed a multicomponent intervention to support AET adherence comprising: text messages, information leaflet, acceptance and commitment therapy (ACT), and side-effect website. Guided by the multiphase optimization strategy, the intervention components were tested in the ROSETA pilot optimization trial. Our mixed-methods process evaluation investigated component acceptability. The pilot optimization trial used a 2^4–1^ fractional factorial design. Fifty-two women prescribed AET were randomized to one of eight experimental conditions, containing unique component combinations. An acceptability questionnaire was administered 4 months post-randomization, and semi-structured interviews with 20 participants further explored acceptability. Assessments were guided by four constructs of the theoretical framework of acceptability: affective attitude, burden, perceived effectiveness, and coherence. Quantitative and qualitative findings were triangulated to identify agreements/disagreements. There were high overall acceptability scores (median = 14–15/20, range = 11–20). There was agreement between the qualitative and quantitative findings when triangulated. Most participants “liked” or “strongly liked” all components and reported they required low effort to engage in. Between 50% (leaflet) and 65% (SMS) “agreed” or “strongly agreed,” it was clear how each component would help adherence. Perceived effectiveness was mixed, with 35.0% (text messages) to 55.6% (ACT) of participants “agreeing” or “strongly agreeing” that each component would improve their adherence. Interview data provided suggestions for improvements. The four components were acceptable to women with breast cancer and will be refined. Mixed-methods and triangulation were useful methodological approaches and could be applied in other optimization trial process evaluations.

**Supplementary Information:**

The online version contains supplementary material available at 10.1007/s11121-024-01711-9.

## Introduction

Women with early-stage (I to III) hormone-receptor-positive breast cancer are prescribed adjuvant endocrine therapy (AET) for 5–10 years to reduce the risk of breast cancer recurrence and mortality (Early Breast Cancer Trialists Collaborative Group, 2011, 2015). However, non-adherence is present in up to three-quarters of women (Hershman et al., 2010; Murphy et al., 2012), which increases the risk of recurrence and reduces quality-adjusted life years (Inotai et al., 2021; Makubate et al., 2013; McCowan et al., 2013). The most recent meta-analysis of interventions, including 25 unique studies to support AET adherence, found an overall significant effect on adherence (Bright et al., 2023). However, several limitations were identified: the frequent use of educational interventions that are unlikely to be sufficient to change behavior alone, the limited use of theory in intervention development, and the lack of focus on key barriers to adherence. There is limited understanding of which strategies can most effectively support AET adherence, with two exceptions: side-effect management education has been largely ineffective, and lowering medication costs has been consistently effective (Bright et al., 2023).

As part of the “Refining and Optimizing a behavioral intervention to Support Endocrine Therapy Adherence” (ROSETA) program, we developed a conceptual model including four theory-informed intervention components that aimed to target key barriers to AET adherence (Green et al., 2022). ROSETA is guided by the Multiphase Optimization Strategy (MOST), an engineering-inspired framework to optimize multicomponent behavioral interventions (Collins, 2018). In the preparation and evaluation phases of MOST, intervention components are typically developed and tested for feasibility and evaluated as a package against a suitable comparator, often using a parallel group randomized controlled trial (RCT) (Collins, 2018). The MOST framework advocates for an additional optimization phase between preparation and evaluation. In this optimization phase, efficient, fully powered experimental designs are used to estimate the main and interaction effects of intervention components (Collins, 2018). These effect estimates can be used to build an optimal intervention package within set constraints, such as time or cost (Collins, 2018; Collins et al., 2021; Strayhorn et al., 2023). The optimization phase aims to balance the effectiveness of an intervention with affordability, scalability, and efficiency.

In the preparation phase of MOST, we conducted an external, multi-center exploratory pilot optimization trial, using a 2^4–1^ fractional factorial design, to pilot procedures and test the feasibility of undertaking a full optimization-randomized controlled trial (ISRCTN: 10487576) (Collins, 2018; Collins et al., 2021; Smith et al., 2023). A fractional factorial design was chosen to halve the number of experimental conditions, which reduced the resources required to set up the experimental conditions and increased the number of participants randomized to each condition. As decision-making about an optimal intervention was not an aim of the pilot trial, aliasing of effects that occur in a fractional factorial design was not considered problematic. Participants were randomized to one of eight experimental conditions which determined the unique combination of components they received, in addition to usual care. Each intervention component had two factor levels: “on” or “off” (Table 1). Fifty-two adult women with stage I-IIIa breast cancer taking AET across five UK hospital sites were randomized. Progression to a full optimization trial is based on criteria regarding consent rates, intervention component adherence, and availability of outcome data (Smith et al., 2023). Detailed methods and results of the main objectives of the pilot optimization trial, relating to feasibility, will be reported elsewhere (Smith et al., 2023).

**Table 1:** Experimental conditions in ROSETA pilot trial

Condition	Usual care	SMS	Information leaflet	ACT	Website	Randomized, *n* = 52	Interviewed, *n* = 20
**1**	Yes	Yes	Yes	Yes	Yes	8	1
**2**	Yes	Yes	Yes	No	No	7	4
**3**	Yes	Yes	No	Yes	No	7	3
**4**	Yes	Yes	No	No	Yes	6	2
**5**	Yes	No	Yes	Yes	No	6	3
**6**	Yes	No	Yes	No	Yes	6	1
**7**	Yes	No	No	Yes	Yes	6	3
**8**	Yes	No	No	No	No	6	3

*ROSETA* Refining and Optimizing a behavioral intervention to Support Endocrine Therapy Adherence, *SMS* short message service, *ACT* acceptance and commitment therapy

Medical Research Council guidance for developing and evaluating complex interventions and process evaluations suggests assessing acceptability in the feasibility stage of intervention development (Moore et al., 2015; Skivington et al., 2021). During the feasibility phase, quantitative and qualitative assessments of acceptability can inform potential adaptations and improvements to intervention components prior to further evaluation (Moore et al., 2015; Sekhon et al., 2017, 2022). Improving acceptability is beneficial at this stage, as greater adherence is more likely with an acceptable intervention (Sekhon et al., 2017). In this process evaluation of the ROSETA pilot optimization trial, we assessed the acceptability of the four intervention components, to identify any necessary adaptions prior to further evaluation.

## Methods

### Design

We used quantitative and qualitative methods to assess the acceptability of each intervention component, guided by the theoretical framework of acceptability (TFA), which defines acceptability as being composed of seven constructs (Sekhon et al., 2017). The seven constructs are (1) affective attitude, *how an individual feels about the intervention*; (2) burden, *perceived amount of effort required to participate*; (3) ethicality, *extent to which the intervention fits with an individual’s values*; (4) coherence, *the extent to which the participant understands the intervention*; (5) opportunity costs, *the extent to which benefits, profits, or values must be given up to engage in the intervention*; (6) perceived effectiveness, *the extent to which the intervention is perceived as likely to achieve its purpose by participant’s who have received it (this is not an assessment of actual effectiveness of the intervention components)*; and (7) self-efficacy, *the participant’s confidence that they can perform the behavior(s) required* (Sekhon et al., 2017).

For the quantitative assessment, all trial participants were invited to complete an adapted version of the acceptability questionnaire 4 months after they were randomized to an experimental condition (4 months post-randomization) (Sekhon et al., 2022). The qualitative assessment involved a semi-structured interview with a sub-sample of trial participants, which took place at least 4 months post-randomization. The interview focused on the acceptability of the intervention components, in addition to fidelity and trial experience related to the wider aims of the process evaluation (Green et al., 2023). As an additional indicator of acceptability, withdrawals from intervention components were recorded, together with the reason for withdrawal (where available).

### Intervention Components

The four intervention components were (1) SMS messages to target forgetfulness, (2) information leaflet to increase beliefs about the necessity of AET and reduce concerns, (3) acceptance and commitment therapy (ACT)-based guided self-help to increase psychological flexibility and reduce psychological distress, and (4) self-management website to support the management of AET side-effects (Table 2). The development of the intervention targets, components, and conceptual model is reported elsewhere (Green et al., 2022). The conceptual model for the intervention is included in Online Resource 1.

**Table 2:** Summary of intervention components in the ROSETA pilot trial

Component	Target	Description
SMS	Forgetfulness/habit formation	SMS messages were sent over 4 months providing practical strategies to support regular medication taking each day. The messages were sent daily for 2 weeks, twice weekly for 8 weeks, and weekly for 6 weeks.
Information leaflet	Medication beliefs	A written information leaflet containing 5 elements: an explanation of how AET works with diagrams to supplement, visual displays of the benefits of AET, accurate information about the side-effects of AET, answers to common concerns about AET, and quotes and pictures of breast cancer survivors.
ACT	Psychological flexibility/psychological distress	A guided self-help intervention based on ACT principles involving four skills: mindfulness, unhooking, following values, and living beyond labels. The modules consist of a participant booklet, home practice tasks, and audio files. The modules are supported by five individual sessions with a psychologist: 1 × 15 min opening session; 3 × 25 min sessions following modules 1, 2, and 3; and 1 × 15 min closing session following module 4.
Website	Side-effect self-management	A website containing strategies to self-manage common AET side-effects including arthralgia, fatigue, vulvovaginal symptoms, gastrointestinal symptoms, hot flushes, and sleep difficulties. The website uses a rating system to summarize the strength of evidence for each strategy.

This table is taken, with permission, from Green et al. (2023)

*ROSETA* Refining and Optimizing a behavioral intervention to Support Endocrine Therapy Adherence, *SMS* short message service, *AET* adjuvant endocrine therapy, *ACT* acceptance and commitment therapy

### Participants

Participants were recruited from five UK NHS hospitals. All participants were women, over 18, taking AET (tamoxifen, raloxifene, anastrozole, letrozole, or exemestane) for early-stage (I to IIIa) breast cancer who had completed their last hospital treatment in the previous 12 months. Full eligibility criteria and recruitment methods are available in the published protocol (Smith et al., 2023).

### Procedure

#### Quantitative Assessment Measures

A validated acceptability questionnaire (AQ) based on the TFA was used to assess intervention component acceptability (Sekhon et al., 2022). To reduce participant burden, we removed three constructs from the TFA (ethicality, self-efficacy, and opportunity cost) we deemed less relevant. This decision was based on a previous similar study investigating the acceptability of an ACT intervention in women with breast cancer, where these constructs were mentioned less frequently in semi-structured interviews (Smith et al., 2022). The remaining four constructs (affective attitude, burden, perceived effectiveness, and intervention coherence) were assessed via four items, with an additional item asking about the general acceptability of each component (e.g., “how acceptable were the SMS messages?”). Participants answered on a five-point Likert scale, with higher scores indicating greater acceptability for all items except for burden, whereby a lower score indicated greater acceptability.

All participants were sent an online questionnaire at 4 months post-randomization. Non-respondents were prompted after 1 and 2 weeks. Participants were given a separate AQ specific to each intervention component they were randomized to receive. For the ACT component, participants were asked 15 extra items about elements of the ACT component (e.g., support sessions, home practice tasks). For the SMS component, participants were asked one extra item regarding the frequency of messages.

#### Qualitative Interviews

All participants willing to be contacted about an interview were emailed with further information and a consent form approximately 3 months post-randomization, to enable the interview to be conducted as close as possible to 4 months post-randomization. Non-respondents were prompted via phone and/or email after 1 week. Participants provided written or telephone consent for the interview.

Semi-structured interviews investigated the acceptability of each intervention component relating to the same four TFA constructs used in the quantitative assessment: affective attitude, burden, perceived effectiveness, and intervention coherence (Sekhon et al., 2017). The interview schedule was developed with input from four women with experience of taking AET (available at 10.17605/OSF.IO/8DWRN). The interview schedule was used as a guide, with flexibility in the order of questions asked and follow-up questions, guided by participant responses. All interviews were conducted via telephone or Microsoft Teams and were recorded either using an encrypted Dictaphone or inbuilt recording software. Interviews took place between December 2022 and April 2023. All interviews were conducted by a researcher (SG) with experience in conducting qualitative interviews.

Due to the digital nature of the intervention components, we aimed to interview a mix of participants above and below 50 years old. We planned to cease interviewing once the sample held sufficient information power: a concept which suggests data collection should stop when the data is sufficiently “information-rich” (Malterud et al., 2016). Continuation of data collection was discussed at regular team meetings. As the number of participants recruited to the ROSETA pilot trial was lower than expected (80 planned, 52 participants randomized, due to a limited recruitment period and low volume of patients eligible to be approached), sampling was opportunistic, as we invited all consenting participants to be interviewed.

#### Data Analysis

A quantitative analysis plan was pre-specified prior to qualitative analyses commencing. Qualitative analyses were completed before quantitative analyses began, both led by one author (SG).

### Qualitative Analysis

We used a rapid qualitative analysis approach to allow findings to be communicated quickly to inform the next phase of the research (Vindrola-Padros et al., 2022). The TFA guided our deductive approach to analysis. The interviewer (SG) took notes during each interview and completed a rapid assessment procedure (RAP) sheet for each participant following the interview (Vindrola-Padros et al., 2021, 2022). The RAP sheet was a three-column table; TFA constructs were included in the first column (in addition to fidelity domains relevant to the wider process evaluation (Green et al., 2023)), relevant notes for each construct were inputted in the second column, and illustrative quotes in the third column (Online Resource 2). For interviews taking place on Microsoft Teams, quotes were taken directly from the inbuilt transcript. For telephone interviews recorded with a Dictaphone, one author (SG) transcribed specific sections of the interview considered important to the research question.

Throughout the data collection period, members of the research team (SG, SS, LH, and CG) met monthly (approximately after 4–5 new interviews had taken place) for the purpose of rapid qualitative analysis. We discussed key findings, adaptations to be made to the intervention components, and any areas to prioritize and explore in upcoming interviews. Individual RAP sheets were collated into four higher-level RAP sheets, whereby one RAP sheet collated all findings for one intervention component. Key findings from the higher-level RAP sheets were summarized.

### Quantitative Analysis

We used descriptive statistics to summarize each individual construct on the AQ, and additional ACT and SMS items. An overall acceptability score was calculated by summing items relating to the TFA constructs affective attitude, burden (reverse coded), perceived effectiveness, and coherence. Missing data were summarized descriptively and were not included in the overall acceptability score calculation.

### Triangulation of Quantitative and Qualitative Findings

Once qualitative and quantitative analyses were complete, findings were triangulated (O'Cathain et al., 2010; Tonkin-Crine et al., 2015). Quantitative findings were summarized into qualitative statements by one author (SG) to aid comparison with qualitative findings. For each of the four TFA constructs (affective attitude, burden, perceived effectiveness, and coherence), key findings from the quantitative and qualitative data were compared for each intervention component. The relationship between the qualitative and quantitative data was marked as either silence (only one data set contained information on a topic), dissonant (conflicting findings), partial agreement (datasets provide complementary findings on a topic), or agreement (full convergence in the data). Two authors (SG and KL) triangulated the findings independently and resolved any disagreements through discussion.

## Results

A total of 141 patients were eligible, of which 52 (36.9%) participants were randomized in the ROSETA pilot trial (Table 1). Reasons for eligible participants not participating included being unwilling, declining, and being unable to contact. Participants had a mean age of 55.2 (SD = 10.8), most (86.5%) were of White ethnicity, and a third (32.7%) had degree level education or above (Table 3). Twenty-one (42.0%) participants had stage I breast cancer, 23 (46.0%) had stage II breast cancer, and 6 (12.0%) had stage IIIA breast cancer. Of the 52 participants, 28 were randomized to receive the SMS component, 27 to the information leaflet, 27 to the ACT component, and 26 to the website (Table 1). Rates of completion for the AQs were 71.4% (*n* = 20) for the SMS component, 74.1% (*n* = 20) for the information leaflet, 70.4% (*n* = 19) for the ACT component, and 73.1% (*n* = 19) for the website. The quantitative assessment of acceptability for each intervention component is summarized in Table 4.

**Table 3:** Participant demographics

		Component				
	Overall, *n* = 52	SMS, *n* = 28	Leaflet, *n* = 27	ACT, *n* = 27	Website, *n* = 26	Interview sample, *n* = 20
Age, mean (SD)	55.2 (10.8)	52.5 (12.4)	56.1 (12.1)	55.4 (11.0)	54.1 (12.0)	57.7 (8.34)
Marital status, n (%)						
Married	32 (61.5)	16 (57.1)	16 (59.3)	17 (63.0)	15 (15.7)	16 (80.0)
Single	6 (11.5)	3 (10.7)	3 (11.1)	3 (11.1)	3 (11.5)	2 (10.0)
Living with a partner	5 (9.6)	4 (14.3)	2 (7.4)	3 (11.1)	3 (11.5)	1 (5.0)
Divorced or separated	7 (13.5)	4 (14.3)	5 (18.5)	3 (11.1)	4 (15.4)	1 (5.0)
Widowed	2 (3.8)	1 (3.6)	1 (3.7)	1 (3.7)	1 (3.8)	0 (0.0)
Employment status, *n* (%)						
Full time	22 (42.3)	9 (32.1)	9 (33.3)	13 (48.1)	9 (34.6)	9 (45.0)
Part time	9 (17.3)	7 (25.0)	6 (22.2)	2 (7.4)	3 (11.5)	4 (20.0)
Not currently working	9 (17.3)	5 (17.9)	3 (11.1)	6 (22.2)	6 (23.1)	1 (5.0)
Other	12 (23.1)	7 (25.0)	9 (33.3)	6 (22.2)	8 (30.8)	6 (30.0)
Education, *n* (%)						
Postgraduate qualification	7 (13.5)	5 (17.9)	5 (18.5)	4 (14.8)	4 (15.4)	3 (15.0)
Degree level education	10 (19.2)	7 (25.0)	4 (14.8)	4 (14.8)	3 (11.5)	6 (30.0)
Higher educational qualifications (below degree)	12 (23.1)	5 (17.9)	6 (22.2)	7 (25.9)	6 (23.1)	6 (30.0)
Vocational qualifications (NVQ1+2)	6 (11.5)	3 (10.7)	4 (14.8)	3 (11.1)	4 (15.4)	0 (0.0)
A-level or equivalent	5 (9.6)	2 (7.1)	3 (11.1)	2 (7.4)	3 (11.5)	1 (5.0)
GCSE/ O-Level/CSE	11 (21.2)	6 (21.4)	5 (18.5)	6 (22.2)	5 (19.2)	4 (20.0)
No formal qualifications	1 (1.9)	0 (0.0)	0 (0.0)	1 (3.7)	1 (3.8)	0 (0.0)
Ethnicity, *n* (%)						
White British	43 (82.7)	25 (89.3)	22 (81.5)	23 (85.2)	20 (76.9)	19 (95.0)
White Irish	1 (1.9)	0 (0.0)	0 (0.0)	1 (3.7)	1 (3.8)	1 (5.0)
Any other white background	1 (1.9)	0 (0.0)	1 (3.7)	0 (0.0)	1 (3.8)	0 (0.0)
Mixed-White and Black Caribbean	1 (1.9)	1 (3.6)	1 (3.7)	0 (0.0)	0 (0.0)	0 (0.0)
Mixed-White and Black African	1 (1.9)	0 (0.0)	1 (3.7)	0 (0.0)	1 (3.8)	0 (0.0)
Asian/Asian British-Indian	1 (1.9)	1 (3.6)	1 (3.7)	1 (3.7)	1 (3.8)	0 (0.0)
Asian/Asian British-Chinese	1 (1.9)	0 (0.0)	0 (0.0)	1 (3.7)	1 (3.8)	0 (0.0)
Black/Black British-Caribbean	2 (3.8)	0 (0.0)	1 (3.7)	1 (3.7)	0 (0.0)	0 (0.0)
Black/Black British-African	1 (1.9)	1 (3.6)	0 (0.0)	0 (0.0)	1 (3.8)	0 (0.0)
Number of children, *n* (%)						
0	10 (19.2)	7 (25.0)	7 (25.9)	6 (22.2)	8 (30.8)	1 (5.0)
1	8 (15.4)	4 (14.3)	5 (18.5)	3 (11.1)	6 (23.1)	3 (15.0)
2	23 (44.2)	12 (42.9)	10 (37.0)	13 (48.1)	9 (34.6)	9 (45.0)
3	9 (17.3)	4 (14.3)	4 (14.8)	4 (14.8)	2 (7.7)	7 (35.0)
4	2 (3.8)	1 (3.6)	1 (3.7)	1 (3.7)	1 (3.8)	0 (0.0)
Stage of diagnosis, *n* (%)						
Stage IA	19 (38.0)	8 (30.8)	12 (44.4)	9 (36.0)	7 (26.9)	7 (35.0)
Stage IB	2 (4.0)	0 (0.0)	1 (3.7)	1 (4.0)	0 (0.0)	2 (10.0)
Stage IIA	15 (30.0)	11 (42.3)	7 (25.9)	7 (28.0)	11 (42.3)	5 (25.0)
Stage IIB	8 (16.0)	4 (15.4)	2 (7.4)	4 (16.0)	4 (15.4)	4 (20.0)
Stage IIIA	6 (12.0)	3 (11.5)	5 (18.5)	4 (16.0)	4 (15.4)	2 (10.0)
Missing^a^	2	2	0	2	0	0
Tumor type, *n* (%)						
Primary	52 (100.0)	28 (100.0)	27 (100.0)	27 (100.0)	26 (100.0)	20 (100.0)
Year of diagnosis, *n* (%)						
2020	3 (5.8)	3 (10.7)	1 (3.7)	1 (3.7)	3 (11.5)	0 (0.0)
2021	33 (63.5)	18 (64.3)	16 (59.3)	20 (74.1)	18 (69.2)	13 (65.0)
2022	16 (30.8)	7 (25.0)	10 (37.0)	6 (22.2)	5 (19.2)	7 (35.0)
Treatment received, *n* (%)						
Surgery: lumpectomy	43 (82.7)	23 (82.1)	23 (85.2)	26 (96.3)	20 (76.9)	18 (90.0)
Surgery: unilateral mastectomy	5 (9.6)	3 (10.7)	1 (3.7)	0 (0.0)	4 (15.4)	1 (5.0)
Surgery: double mastectomy	2 (3.8)	1 (3.6)	2 (7.4)	1 (3.7)	2 (7.7)	0 (0.0)
Neoadjuvant chemotherapy	5 (9.6)	4 (14.3)	3 (11.1)	4 (14.8)	5 (19.2)	1 (5.0)
Adjuvant chemotherapy	18 (34.6)	10 (35.7)	8 (29.6)	13 (48.1)	9 (34.6)	3 (15.0)
Adjuvant radiotherapy	43 (82.7)	23 (82.1)	22 (81.5)	23 (85.2)	20 (76.9)	17 (85.0)
Monoclonal antibody-based therapy	4 (7.7)	1 (3.6)	1 (3.7)	3 (11.1)	3 (11.5)	1 (5.0)
Other	13 (25.0)	8 (28.6)	8 (29.6)	7 (25.9)	3 (11.5)	5 (25.0)
Current hormone therapy, *n* (%)						
Tamoxifen	12 (23.1)	9 (32.1)	5 (18.5)	7 (25.9)	5 (19.2)	8 (40.0)
Anastrozole	8 (15.4)	3 (10.7)	4 (14.8)	6 (22.2)	3 (11.5)	1 (5.0)
Exemestane	3 (5.8)	1 (3.6)	2 (7.4)	1 (3.7)	2 (7.7)	1 (5.0)
Letrozole	29 (55.8)	15 (53.6)	16 (59.3)	13 (48.1)	16 (61.5)	10 (50.0)
Menopausal status, *n* (%)						
Pre-menopausal	12 (23.1)	10 (35.7)	8 (29.6)	5 (18.5)	7 (26.9)	2 (10.0)
Peri-menopausal	3 (5.8)	2 (7.1)	0 (0.0)	2 (7.4)	2 (7.7)	3 (15.0)
Post-menopausal	30 (57.7)	11 (39.3)	17 (63.0)	15 (55.6)	15 (57.7)	11 (55.0)
Unsure	7 (13.5)	5 (17.9)	2 (7.4)	5 (18.5)	2 (7.7)	4 (20.0)

All clinical data was completed by the site

*SMS* short message service, *ACT* acceptance and commitment therapy

^a^Missing data was not included in percentage calculations

**Table 4:** Acceptability questionnaire scores per component

Acceptability construct	Intervention components			
	SMS, *n* = 20	Information leaflet, *n* = 20	ACT, *n* = 19	Website, *n* = 19
Overall acceptability, median (range)	14 (11–20)	14.5 (12–17)	15 (11–19)	15 (12–20)
General acceptability, *N* (%)				
Completely unacceptable	0 (0.0)	1 (5.0)	2 (11.1)	0 (0.0)
Unacceptable	0 (0.0)	0 (0.0)	0 (0.0)	0 (0.0)
No opinion	1 (5.0)	4 (20.0)	1 (5.6)	5 (26.3)
Acceptable	11 (55.0)	9 (45.0)	3 (16.7)	6 (31.6)
Completely acceptable	8 (40.0)	6 (30.0)	12 (66.7)	8 (42.1)
Missing	0	0	1	0
Affective attitude, *N* (%)				
Strongly dislike	0 (0.0)	0 (0.0)	0 (0.0)	0 (0.0)
Dislike	1 (5.0)	0 (0.0)	0 (0.0)	0 (0.0)
No opinion	8 (40.0)	9 (45.0)	2 (11.1)	5 (26.3)
Like	10 (50.0)	10 (50.0)	5 (27.8)	9 (47.4)
Strongly like	1 (5.0)	1 (5.0)	11 (61.1)	5 (26.3)
Missing	0	0	1	0
Burden, *N* (%)				
No effort at all	11 (55.0)	10 (50.0)	1 (5.6)	6 (31.6)
A little effort	6 (30.0)	5 (25.0)	10 (55.6)	8 (42.1)
No opinion	3 (15.0)	4 (20.0)	1 (5.6)	5 (26.3)
A lot of effort	0 (0.0)	1 (5.0)	3 (16.7)	0 (0.0)
Huge effort	0 (0.0)	0 (0.0)	3 (16.7)	0 (0.0)
Missing	0	0	1	0
Perceived effectiveness, *N* (%)				
Strongly disagree	3 (15.0)	1 (5.0)	1 (5.6)	0 (0.0)
Disagree	3 (15.0)	0 (0.0)	1 (5.6)	4 (21.5)
No opinion	7 (35.0)	11 (55.0)	6 (33.3)	8 (42.1)
Agree	6 (30.0)	8 (40.0)	5 (27.8)	6 (31.6)
Strongly agree	1 (5.0)	0 (0.0)	5 (27.8)	1 (5.3)
Missing	0	0	1	0
Coherence, *N* (%)				
Strongly disagree	0 (0.0)	0 (0.0)	1 (5.6)	0 (0.0)
Disagree	2 (10.0)	0 (0.0)	3 (16.7)	1 (5.3)
No opinion	5 (25.0)	10 (50.0)	4 (22.2)	8 (42.1)
Agree	11 (55.0)	10 (50.0)	6 (33.3)	8 (42.1)
Strongly agree	2 (10.0)	0 (0.0)	4 (22.2)	2 (10.5)
Missing	0	0	1	0

Only data from participants who completed the acceptability questionnaires were included. Percentages were calculated excluding missing data

*SMS* short message service, *ACT* acceptance and commitment therapy

Overall, 46 (88.5%) participants consented to be approached for interview. Of these, 5 withdrew from the trial and the remaining 41 participants were invited for interview. A total of 20 (48.8% of those invited) participants were interviewed, 6 declined (14.6%), and 15 (36.6%) did not respond. Of the 20 participants interviewed, 10 participants received the SMS component, 9 received the information leaflet, 10 received the ACT component, and 7 received the website (Table 1). Three interviewed participants were from condition eight; as they did not receive any intervention components, their data did not contribute to the analysis. The interviews took place between 0 and 46 days after the 4-month follow-up questionnaire was sent out and lasted between 11 and 62 min. The interview sample held sufficient information power to determine the acceptability of the four intervention components (Malterud et al., 2016). A summary of the key findings from the interviews in terms of the acceptability of each intervention component is displayed in Table 5. In triangulation, 38 comparisons were made between the quantitative and qualitative findings (Table 6). There were 13 disagreements between the coders which were resolved via discussion.

**Table 5:** Summary of rapid qualitative analysis of each intervention component across constructs of the theoretical framework of acceptability

Acceptability construct	SMS	Leaflet	ACT	Website
Affective attitude	• Most women felt the messages were a good idea and found the content interesting and informative • A minority felt some messages were too much like common sense or out of place	• Several aspects of the leaflet were liked, including the quotes from other women, and information about side-effects • One participant felt they already knew the information but liked having the information written down	• Participants liked the practical, skills focus • A number of ACT skills were liked and applied. Examples included using mindfulness to reduce hot flushes and identifying values to get back to enjoyed activities such as volunteering • Support sessions from the therapist were liked by all participants overall • Most participants felt the timing of the sessions were good, as other support had ceased. One participant felt they were not ready for the sessions • One participant felt some pressure to talk in the sessions to fill the time	• Some women felt it was beneficial to see videos of what other women are experiencing. However, one participant felt the videos were too stereotypical • One participant felt the website was not aesthetically pleasing • A few participants found the information too general and vague in places • Some women liked the honesty of the evidence ratings for the side-effect management strategies, but others did not feel this was helpful
Burden	• Overall low burden and not intrusive • Two participants felt daily messages were too frequent • Some participants may have opted out if not within trial setting	• Many women felt the leaflet was concise and easy to read, without “medical jargon”	• Most participants liked the online delivery and flexibility of sessions • Weekly sessions too close together—need more time to practice skills • Therapy is emotionally challenging—having sessions in the morning and then going back to work was difficult	• The website modality was acceptable
Coherence	• The majority of women understood the messages were about building routines of taking medication • Some women felt the messages were a prompt to take medication • Some felt the messages emphasized the importance of taking medication • One participant felt the messages were a form of social support	• Most women understood the leaflet was aiming to provide information about AET	• Overall understanding that ACT was teaching skills and coping mechanisms to move forwards • Some participants were unsure about how ACT would help them to adhere to AET when beginning the intervention but gained more understanding after attending a few sessions	• Participants generally understood the website was to provide side-effect self-management strategies
Perceived effectiveness	• Most women felt they had routines to take AET but that the messages would be effective for those who did not • Some women felt personalizing the timing of the messages would make them more beneficial	• Some women reported being able to go back to the leaflet and re-read it to remind themselves of the benefits was helpful to remind them why they are taking AET	Multiple experiences were shared regarding perceived effectiveness: • How ACT had helped take AET • Reduced psychological distress • Helped to return to work • Helped to cope with side-effects of AET	• Some women acknowledged the website would be helpful for those experiencing side-effects, who have not researched coping strategies • Some women felt the website did not teach them anything new
Other		• A number of women could not recall receiving the leaflet		• Some women could not recall receiving website login details

*SMS* short message service, *ACT* acceptance and commitment therapy, *AET* adjuvant endocrine therapy

**Table 6:** Triangulation of quantitative and qualitative findings

Component	Triangulation	TFA construct				
		Affective attitude	Burden	Perceived effectiveness	Coherence	Total
SMS	Silence	0	2	1	0	3
	Dissonance	0	0	0	0	0
	Partial agreement	2	2	1	4	9
	Agreement	0	2	0	0	2
Leaflet	Silence	0	0	0	0	0
	Dissonance	0	0	0	0	0
	Partial agreement	2	1	1	1	5
	Agreement	0	0	0	0	0
ACT	Silence	0	1	0	0	1
	Dissonance	1	0	0	0	1
	Partial agreement	4	2	1	2	9
	Agreement	0	0	0	0	0
Website	Silence	0	1	0	0	1
	Dissonance	3	0	0	0	3
	Partial agreement	1	0	2	1	4
	Agreement	0	0	0	0	0

*SMS* short message service, *ACT* acceptance and commitment therapy

### Overall Acceptability

All intervention components were considered acceptable, with overall acceptability scores ranging between 14/20 (SMS) and 15/20 (ACT and website), across components (range 11–20). For all components, most participants rated each TFA construct at the midpoint or above (Table 4).

### SMS

In the quantitative assessment, 19 out of 20 (95.0%) participants reported the SMS messages were “acceptable” or “completely acceptable” (*general acceptability*) (Table 4). The burden was low, with no participants reporting the SMS messages were “a lot of effort,” or a “huge effort” to engage with. Seven (35.0%) participants “agreed” or “strongly agreed” that the SMS messages would help them take AET, and a further seven (35.0%) had “no opinion” (*perceived effectiveness*). Thirteen participants “agreed” or “strongly agreed” that it was clear how the messages would help them to take AET (*coherence*). Two of the seven participants who withdrew/opted-out from the SMS component cited dislike of the SMS messages as their reason for withdrawal (Online Resource 3). Most participants (18/20, 90.0%) reported the frequency of SMS messages was “acceptable” or “completely acceptable” (Online Resource 4).

In the interviews, participants reported that overall, they liked the SMS messages (*affective attitude*) (Table 5, Online Resource 5)*.* Most participants reported they already had routines in place to take their medication and so did not feel the messages would have provided additional benefit to them, but acknowledged the potential effectiveness among women who may not have such routines (*perceived effectiveness*). No women interviewed opted out of receiving the messages, and only a minority felt the daily messages were too frequent (*burden*). Most participants understood the intended target for the messages, in that they were aiming to build routines in taking medication. Some women also perceived the aims to be to prompt daily medication taking, to emphasize the importance of taking medication, and to provide social support (*coherence*).

A total of 14 comparisons were made between the quantitative and qualitative data for triangulation of the SMS component. Most comparisons observed partial agreement (Table 6). There were three instances of silence, in which the qualitative data provided data on a topic that the quantitative data did not refer to, such as suggested improvements to the timing of the SMS messages (Online Resource 6).

### Information Leaflet

Of the 20 participants who completed the AQ, 15 (75.0%) found the leaflet “acceptable” or “completely acceptable” (*general acceptability*), and the majority (15, 75.0%) felt it was “no effort at all” or “a little effort” to read (*burden*) (Table 4). Eleven out of 20 (55.0%) participants “liked” or “strongly liked” the leaflet (*affective attitude*), while nine (45.0%) had “no opinion.” Eight (40.0%) participants “agreed” that the leaflet would help them to take AET, but 11 (55.0%) had “no opinion” (*perceived effectiveness*). Half the participants “agreed” it was clear how the leaflet would help them take AET, while the other half had “no opinion” (*coherence*) (Table 4).

In the interviews, participants reported liking aspects of the information leaflet, including the quotes from other women with breast cancer, information about AET side-effects, and clear information about the benefits of AET (*affective attitude*). However, several women randomized to receive the leaflet could not recall receiving it, often explaining that they received a lot of information at once regarding the trial. When asked about the perceived effectiveness of the leaflet, some women reflected on the usefulness being that they could re-read the leaflet to remind themselves why they were taking AET (*perceived effectiveness*) (Table 5, Online Resource 5)*.* Five comparisons were made for the triangulation of the leaflet (Table 6). All comparisons were coded as partial agreement, with the qualitative data adding context to the quantitative data (Online Resource 6).

### ACT

Of the 27 participants randomized to receive the ACT component, 24 (88.9%) attended session one, 21 (77.8%) attended session, two 17 (63.0%) attended session three, 17 (63.0%) attended session four, and 16 (59.3%) attended session five. Of the eight participants who withdrew from the ACT component, only one cited dislike of the ACT component as the reason for withdrawal (Online Resource 3).

Of the participants who completed the ACT AQ, 15 (83.4%) felt the ACT component was “acceptable” or “completely acceptable” (*general acceptability*). Most (16, 88.9%) participants “liked” or “strongly liked” the ACT component (*affective attitude*). The burden was mixed; 11 (61.2%) participants felt engaging in the ACT sessions was “no effort at all,” or “a little effort,” 1 (5.6%) participant had “no opinion,” and 6 (33.4%) felt it was “a lot of effort” or “a huge effort.” Ten (55.6%) participants “agreed” or “strongly agreed” that the ACT component would help them to take AET (*perceived effectiveness*) and that it was clear how the ACT component would help them to take their AET (*coherence*). Acceptability of the ACT component overall and individual aspects of the ACT intervention did not vary considerably across the five sites, each with different therapists delivering the intervention (Online Resource 4).

Interviewed participants were enthusiastic about the ACT component overall, citing several ACT skills that they liked, including mindfulness, unhooking, and values-based exercises (*affective attitude*). The participants were positive about their therapeutic relationship, with frequent reports of feeling comfortable opening up and being listened to (*affective attitude*). One participant felt pressure to keep talking to fill the time in the sessions (*affective attitude*). For most participants, the burden of the intervention was perceived to be minimal, made easier through the online delivery and individual nature of sessions allowing flexibility (*burden*). However, one participant acknowledged the emotional burden of attending therapy, and some reported that the weekly sessions were too close together. Many participants reported understanding that the ACT sessions were skills-focused, but a few participants were apprehensive prior to a session as they did not know what to expect or how this was going to help them (*coherence*). When asked about the perceived effectiveness of the ACT component, participants shared numerous experiences of their personal benefits, including improving their mental health, coping with AET side-effects, reducing stress on returning to work, and adhering to AET (*perceived effectiveness*). Many participants felt the timing of the support was beneficial, at a time when other hospital-based support and appointments had ended (Table 5, Online Resource 5).

Eleven comparisons were made for triangulation of the ACT component, with most indicating partial agreement or agreement between the data (Table 6). The one instance of dissonance occurred whereby the qualitative data indicated some dislike of feeling pressure to talk in the sessions, whereas the quantitative data for affective attitude did not indicate any dislike of the component (Online Resource 6).

### Website

Most (14/19, 73.7%) participants who completed the website AQ thought the website was “acceptable” or “completely acceptable” (*general acceptability*) and “liked” or “strongly liked” the website (*affective attitude*). Most participants (14, 73.7%) felt the website was “no effort at all” or “a little effort” to read and the remainder (5, 26.3%) had “no opinion” (*burden*). Around a third of participants (7, 36.8%) “agreed” or “strongly agreed” that the website would help them to take AET, 8 (42.1%) had “no opinion,” and 4 (21.1%) “disagreed” (*perceived effectiveness*). Most (10, 52.6%) participants “agreed” or “strongly agreed” that it was clear how the website would help them take AET, and 8 (42.1%) had “no opinion” (*coherence*).

In the interviews, there were mixed opinions about the website (*affective attitude*). Some women liked aspects of the website, including the videos of other women sharing their experiences of taking AET. However, other women disliked certain aspects, feeling as if the website was not aesthetically pleasing, was not modern enough for younger participants, and that information was too vague in places (*affective attitude*). There were mixed opinions about the evidence ratings of each side-effect self-management strategy; some women liked the honest nature of this, while others felt it could be demotivating for women who are struggling with side-effects. Multiple women felt the website did not teach them anything new but acknowledged that the information could be helpful for women who have not already researched coping strategies (*perceived effectiveness*). Some women could not recall receiving log in details for the website (Table 5, Online Resource 5).

A total of eight comparisons were made for triangulation of the website. There were three instances of dissonance between the data, which related to occasions whereby qualitative findings included some negative comments about the website, whereas the quantitative assessment did not indicate any dislike in the affective attitude construct (Online Resource 6).

## Discussion

This nested mixed-methods process evaluation of a fractional factorial pilot optimization trial demonstrated overall acceptability of four intervention components aiming to support medication adherence to AET in women with breast cancer. We identified key areas of each intervention component that could be adapted to further improve intervention acceptability prior to a larger optimization trial.

Understanding the acceptability of each intervention component had several implications. In response to some participants feeling the ACT component was burdensome, we amended the delivery to fortnightly sessions rather than weekly. Similarly, a choice of time of day to receive the SMS messages will be offered in the full optimization trial, in response to interview data. Due to some indifference toward the information leaflet, and a proportion of women not recalling receiving the leaflet or the website components, we have changed the timing of delivery of both components to 1 week after randomization, to minimize the chance they were lost among other information. A list of key adaptations and rationales for change is available in Online Resource 7. Undertaking this process evaluation provided important insights and an opportunity to make adaptations to improve acceptability.

A mixed-methods approach added value to understanding the acceptability of the components, and triangulation strengthened the conclusions. A high proportion of the data was coded as “partial agreement,” which reflected the qualitative data adding context to the quantitative findings. For example for coherence, we quantitatively assessed whether a participant felt they understood how the component would help them take their AET, but this did not provide insight into whether their understanding matched the intended design of the component. The qualitative data added important context to aid interpretation. The use of quantitative data alone may have led to different interpretations; a mixed-methods approach and triangulation provided a more thorough understanding of acceptability.

Despite only a small proportion of participants reporting negative responses regarding perceived effectiveness, scores for this construct were still lower than other TFA constructs. A more substantial proportion reported “no opinion,” which could in part reflect that many people do not have insight into exactly what changes their behavior. In some cases, the interview data provided useful context to explain the lower perceived effectiveness. For example, women in the SMS component reported no problems remembering to take their medication thereby reducing the need for the SMS messages. However, they acknowledged the messages could be effective for others. Moreover, some women reported finding the ACT component helpful but were not clear how it would impact their adherence. Focusing acceptability on a primary outcome (e.g., adherence) limits considerations of perceived effectiveness on secondary outcomes that may be important to a participant (e.g., reduction in side-effects). Future assessments of acceptability should consider asking about intervention targets or mediators which may be more proximal to participants, rather than focusing solely on the primary outcome.

To some extent, lower perceived effectiveness may be expected, as we investigated individual intervention components, rather than the intervention package. It is logical that the perceived effectiveness of an intervention component may be lower, as we expect multiple components will be needed to impact medication adherence. The more passive, educational components (information leaflet and website) may not be sufficient to change medication adherence behavior alone, and therefore, perceived effectiveness may be lower (Bright et al., 2023). These components are most likely to impact adherence via interactions with other components, which can be empirically estimated using a factorial design. Therefore, we have retained all four intervention components in the planned optimization trial. We will use data from the full optimization trial to make decisions on which combination of the four intervention components best balances effectiveness with efficiency, affordability, and scalability.

In triangulation, there were only four instances of dissonance between qualitative and quantitative data. All instances of dissonance related to the affective attitude TFA construct and reflected some expression of dislike toward the component in the qualitative data, but no indication of dislike in the quantitative data. It is possible that in the qualitative interviews, participants were able to express opinions on finer details of the intervention components, but that these dislikes did not warrant a negative score when completing the quantitative assessments. We made adaptations to the components based on participants’ responses, as detailed in Online Resource 7.

Undertaking a mixed-methods process evaluation of a trial using a fractional factorial design required some key considerations. For participants randomized to receive multiple intervention components, completing an AQ for each component added burden. Investigators considering such an approach should be mindful of this, particularly if assessing four or more intervention components in a 2^ k^ factorial design (Collins, 2018; Collins et al., 2021). The number of experimental conditions added complexity when considering participant sampling for the interviews. We felt it was important to interview at least one participant from each of the eight conditions, as experienced acceptability could differ dependent upon combinations of intervention components. Attempting to interview participants from all eight experimental conditions while purposively sampling across multiple demographics was logistically complex, and therefore, we planned to focus on purposive sampling across age only. If the primary aim is focused on the individual intervention components, sample size may need to be increased for qualitative studies in a factorial trial compared with those in a parallel group RCT. This is because, on average in a factorial trial, half the participants interviewed will have received a component and half will not (Collins, 2018; Collins et al., 2021).

The resource management principle (RMP) is a key principle of the MOST framework that emphasizes the importance of making the best use of resources available (Collins, 2018). The RMP guided our study design, data collection, and analysis. Using a fractional factorial design, rather than a full factorial design, reduced the number of experimental conditions, and hence the resources required to set up an experimental condition (e.g., development of condition-specific study documents). We had a finite time to deliver the pilot trial; the RMP guided our decision to cease data collection before we reached our target of 80 participants, to ensure we had sufficient time to deliver the fully powered optimization trial. Finally, the RMP guided our decision to use rapid qualitative analysis, as we had a short period of time to make adaptations to the intervention components before proceeding with a larger optimization trial (Smith et al., 2023). We saved time by using automatic and selective transcription and commencing analysis after only a few interviews had taken place. This enabled early consideration of improvements to be made to the intervention components, to ensure adaptations could be implemented in the next phase of the research (Vindrola-Padros et al., 2022).

### Limitations

We excluded three less relevant constructs of the TFA in our assessment of acceptability: ethicality, self-efficacy, and opportunity cost. This decision was made to reduce participant burden, as participants were asked to complete an AQ for each intervention component they were randomized to receive. Including all constructs of the TFA could have led to different insights on acceptability. Our sample consisted predominantly of White women, and therefore, we have not captured the acceptability or appropriateness of the intervention components in a more diverse sample, in which acceptability may have differed. In the planned optimization trial, we will seek to recruit hospital sites in more diverse areas and will include an additional self-referral recruitment route to enable targeted advertisement to specific support groups. There may have been some recall bias, as assessments were conducted 4 months post-randomization. Responses may have been slightly positively skewed, as acceptability among the 30% of participants who did not complete the acceptability questionnaire may have been lower. We were also unable to interview participants who withdrew from receiving the intervention components as they were no longer eligible to be contacted, which may have biased the qualitative findings to women who had a more positive experience. However, we have included relevant data on withdrawals and reasons to aid overall understanding of acceptability across all trial participants. One interviewer (SG) conducted all the interviews and was involved in intervention development, which allowed an in-depth assessment of acceptability but may have introduced bias to the interviews. Multiple researchers (SG, LH, SS, CG) attended qualitative analysis meetings, and a researcher independent to the trial team (KL) triangulated the findings in an attempt to reduce bias.

## Conclusions

We have demonstrated the acceptability of four intervention components aimed at supporting medication adherence in women with breast cancer. Using a mixed-methods approach based on the TFA was helpful in providing a detailed assessment of the acceptability of each of the intervention components. Our rapid qualitative approach enabled our findings to be analyzed quickly to inform adaptations of the intervention components for the next phase of this research. We have demonstrated one approach to conducting a process evaluation which could be applied in other pilot optimization trial process evaluations.

## Supplementary Information

Below is the link to the electronic supplementary material.

Online Resource 1 ROSETA conceptual model (PDF 166 KB)

Online Resource 2 Participant rapid assessment procedure sheet (PDF 422 KB)

Online Resource 3 Summary of participant withdrawals from SMS and ACT components (PDF 546 KB)

Online Resource 4 Additional acceptability results for SMS and ACT components (PDF 726 KB)

Online Resource 5 Illustrative quotes to supplement qualitative analysis (PDF 625 KB)

Online Resource 6 Detailed triangulation comparisons (XLSX 29 KB)

Online Resource 7 Intervention component adaptations (DOCX 22 KB)

## Data Availability

Data will only be shared for participants who have given consent to use of their data for secondary research and will only be made available in such a way that recipients cannot identify individuals by any reasonable likely means. Requests to access quantitative data should be made to CTRU-DataAccess@leeds.ac.uk in the first instance. Qualitative data is available on reasonable request by contacting the corresponding author.
