# Supplementary material for: Acceptability of Four Intervention Components Supporting Medication Adherence in Women with Breast Cancer: a Process Evaluation of a Fractional Factorial Pilot Optimization Trial
Source: Prev Sci. 2024 Jul 26;25(7):1065–78. doi: 10.1007/s11121-024-01711-9 (PMC11519312; doi:10.1007/s11121-024-01711-9)
Supplement: Supplementary file 2 — Online Resource 2 Participant rapid assessment procedure sheet (PDF 422 KB) [file 11121_2024_1711_MOESM2_ESM.pdf]

Supplementary material 1: Individual RAP Sheet

SMS Component	Notes	Quotes
Fidelity of receipt (receiving messages, understanding of messages)		
Barriers to receipt		
Opt out and reasons		
Fidelity of enactment (using suggestions from messages)		
Barriers to enactment		
Affective attitude- likes		
Affective attitude- dislikes		
Burden		
Coherence		
Perceived effectiveness		
Improvements		
Miscellaneous		

Article title: Acceptability of four intervention components supporting medication adherence in women with breast cancer: A process evaluation of a fractional factorial pilot optimization trial
Journal name: Prevention Science
Author names: Sophie M. C. Green, Nikki Rousseau, Louise H. Hall, David P. French, Christopher D. Graham, Kelly E. Lloyd, Michelle Collinson, Pei Loo Ow, Christopher Taylor, Daniel Howdon, Robbie Foy, Rebecca Walwyn, Jane Clark, Catherine Parbutt, Jo Waller, Jacqueline Buxton, Sally J. L. Moore, Galina Velikova, Amanda Farrin, Samuel G. Smith
Corresponding author: Sophie M. C. Green. Leeds institute of Health Sciences, University of Leeds. Email: s.m.c.green@leeds.ac.uk

Information Leaflet	Notes	Quotes
Fidelity of receipt (receiving, reading, understanding)		
Barriers to receipt		
Fidelity of enactment (using suggestions)		
Barriers to enactment		
Affective attitude-likes		
Affective attitude-dislikes		
Burden		
Coherence		
Perceived effectiveness		
Improvements		
Miscellaneous		

ACT	Notes	Quotes
Fidelity of receipt (attending sessions, understanding)		
Barriers to receipt		
Fidelity of enactment (using skills)		

Barriers to enactment		
Affective attitude- likes		
Affective attitude- dislikes		
Burden		
Coherence		
Perceived effectiveness		
Relationship with therapist		
Improvements		
Miscellaneous		

Website	Notes	Quotes
Fidelity of receipt (using website, understanding)		
Barriers to receipt		
Fidelity of enactment (using strategies)		
Barriers to enactment		
Affective attitude- likes		
Affective attitude- dislikes		
Burden		
Coherence		

Perceived effectiveness		
Improvements		
Miscellaneous		
