# Supplementary material for: Acceptability of Four Intervention Components Supporting Medication Adherence in Women with Breast Cancer: a Process Evaluation of a Fractional Factorial Pilot Optimization Trial
Source: Prev Sci. 2024 Jul 26;25(7):1065–78. doi: 10.1007/s11121-024-01711-9 (PMC11519312; doi:10.1007/s11121-024-01711-9)
Supplement: Supplementary file 3 — Online Resource 3 Summary of participant withdrawals from SMS and ACT components (PDF 546 KB) [file 11121_2024_1711_MOESM3_ESM.pdf]

Supplementary Material 2- Withdrawals from SMS and ACT components

Table 1. *Withdrawal from SMS and ACT components*

Condition	SMS withdrawal	ACT withdrawal	Time of ACT withdrawal	Reason(s) for withdrawal
1 (SMS, Leaflet, ACT, Web)	Y	Y	Before any ACT sessions	• Discontinuation of ACT due to eligibility violation and had preference for ACT<br>• Does not need help remembering to take medication<br>• Too many SMS messages<br>• Does not like SMS intervention
1 (SMS, Leaflet, ACT, Web)	Y	Y	After ACT session 1	• Does not like the ACT intervention<br>• Changed mind about trial involvement<br>• Computer literacy
1 (SMS, Leaflet, ACT, Web)	Y	Y	After ACT session 1	• Clinical decision
1 (SMS, Leaflet, ACT, Web)	N	Y	After ACT session 4	• Bereavement
2 (SMS, Leaflet)	Y	N/A	N/A	• No reason given
3 (SMS, ACT)	Y	Y	After ACT session 2	• Does not have the time to give to the intervention; not the right time.<br>• Involvement too much as back at work on a phased return.
3 (SMS, ACT)	Y	N	N/A	• Does not need help remembering to take medication
4 (SMS, Web)	Y	N/A	N/A	• Had enough of SMS and questionnaires
4 (SMS, Web)	N/A	N/A	N/A	• Changed mind about trial involvement<br>• Does not feel the study is having any impact on her
5 (Leaflet, ACT)	N/A	Y	After ACT session 1	• Personal issues unrelated to health
5 (Leaflet, ACT)	N/A	Y	After ACT session 2	• No reason given
7 (ACT, Web)	N/A	Y	After ACT session 2	• Changed mind about trial involvement

Key: SMS= Short Message Service. ACT = Acceptance and Commitment Therapy. Web= Website component.

Article title: Acceptability of four intervention components supporting medication adherence in women with breast cancer: A process evaluation of a fractional factorial pilot optimization trial
Journal name: Prevention Science
Author names: Sophie M. C. Green, Nikki Rousseau, Louise H. Hall, David P. French, Christopher D. Graham, Kelly E. Lloyd, Michelle Collinson, Pei Loo Ow, Christopher Taylor, Daniel Howdon, Robbie Foy, Rebecca Walwyn, Jane Clark, Catherine Parbutt, Jo Waller, Jacqueline Buxton, Sally J. L. Moore, Galina Velikova, Amanda Farrin, Samuel G. Smith
Corresponding author: Sophie M. C. Green. Leeds institute of Health Sciences, University of Leeds. Email: s.m.c.green@leeds.ac.uk
