# Supplementary material for: Acceptability of Four Intervention Components Supporting Medication Adherence in Women with Breast Cancer: a Process Evaluation of a Fractional Factorial Pilot Optimization Trial
Source: Prev Sci. 2024 Jul 26;25(7):1065–78. doi: 10.1007/s11121-024-01711-9 (PMC11519312; doi:10.1007/s11121-024-01711-9)
Supplement: Supplementary file 4 — Online Resource 4 Additional acceptability results for SMS and ACT components (PDF 726 KB) [file 11121_2024_1711_MOESM4_ESM.pdf]

Supplementary material 3- SMS and ACT Additional Acceptability Items

Table 1

Additional SMS acceptability item

Frequency of SMS messages	n= 20
Completely unacceptable	0 (0.0)
Unacceptable	1 (5.0)
No opinion	1 (5.0)
Acceptable	14 (70.0)
Completely acceptable	4 (20.0)

Note: Only data from participants who completed the acceptability questionnaires are included.

Key: SMS= Short Message Service.

Article title: Acceptability of four intervention components supporting medication adherence in women with breast cancer: A process evaluation of a fractional factorial pilot optimization trial
Journal name: Prevention Science
Author names: Sophie M. C. Green, Nikki Rousseau, Louise H. Hall, David P. French, Christopher D. Graham, Kelly E. Lloyd, Michelle Collinson, Pei Loo Ow, Christopher Taylor, Daniel Howdon, Robbie Foy, Rebecca Walwyn, Jane Clark, Catherine Parbutt, Jo Waller, Jacqueline Buxton, Sally J. L. Moore, Galina Velikova, Amanda Farrin, Samuel G. Smith
Corresponding author: Sophie M. C. Green. Leeds institute of Health Sciences, University of Leeds. Email: s.m.c.green@leeds.ac.uk

Table 2

Additional ACT acceptability items, overall and by site

	Overall, n=19	Nottingham, n=2	Gateshead, n=4	King's Lynn, n=5	Woolwich, n=5	Whiston, n=3
**Usefulness of ACT components, n (%)**						
**Therapist support sessions**						
Not at all	1 (5.6)	1 (100.0)	0 (0.0)	0 (0.0)	0 (0.0)	0 (0.0)
A little	0 (0.0)	0 (0.0)	0 (0.0)	0 (0.0)	0 (0.0)	0 (0.0)
Somewhat	3 (16.7)	0 (0.0)	1 (25.0)	1 (20.0)	0 (0.0)	1 (33.3)
Very	14 (77.8)	0 (0.0)	3 (75.0)	4 (80.0)	5 (100)	2 (66.7)
Missing	1	1	0	0	0	0
**Participant manual**						
Not at all	1 (5.6)	1 (100.0)	0 (0.0)	0 (0.0)	0 (0.0)	0 (0.0)
A little	0 (0.0)	0 (0.0)	0 (0.0)	0 (0.0)	0 (0.0)	0 (0.0)
Somewhat	4 (22.2)	0 (0.0)	1 (25.0)	1 (20.0)	1 (20.0)	1 (33.3)
Very	13 (72.2)	0 (0.0)	3 (75.0)	4 (80.0)	4 (80.0)	2 (66.7)
Missing	1	1	0	0	0	0
**Home practice exercises**						
Not at all	1 (5.6)	1 (100.0)	0 (0.0)	0 (0.0)	0 (0.0)	0 (0.0)
A little	2 (11.1)	0 (0.0)	1 (25.0)	0 (0.0)	1 (20.0)	0 (0.0)
Somewhat	2 (11.1)	0 (0.0)	0 (0.0)	1 (20.0)	0 (0.0)	1 (33.3)
Very	13 (72.2)	0 (0.0)	3 (75.0)	4 (80.0)	4 (80.0)	2 (66.7)
Missing	1	1	0	0	0	0
**Audio files**						
Not at all	1 (5.6)	1 (100.0)	0 (0.0)	0 (0.0)	0 (0.0)	0 (0.0)
A little	2 (11.1)	0 (0.0)	1 (25.0)	0 (0.0)	1 (20.0)	0 (0.0)
Somewhat	2 (11.1)	0 (0.0)	0 (0.0)	1 (20.0)	1 (20.0)	0 (0.0)
Very	13 (72.2)	0 (0.0)	3 (75.0)	4 (80.0)	3 (60.0)	3 (100.0)
Missing	1	1	0	0	0	0
**Introductory session**						
Not at all	1 (5.6)	1 (100.0)	0 (0.0)	0 (0.0)	0 (0.0)	0 (0.0)
A little	2 (11.1)	0 (0.0)	1 (25.0)	0 (0.0)	1 (20.0)	0 (0.0)
Somewhat	2 (11.1)	0 (0.0)	0 (0.0)	2 (40.0)	0 (0.0)	0 (0.0)
Very	13 (72.2)	0 (0.0)	3 (75.0)	3 (60.0)	4 (80.0)	3 (100.0)
Missing	1	1	0	0	0	0
**Session 2**						
Not at all	1 (5.9)	1 (100.0)	0 (0.0)	0 (0.0)	0 (0.0)	0 (0.0)
A little	1 (5.9)	0 (0.0)	0 (0.0)	0 (0.0)	1 (20.0)	0 (0.0)
Somewhat	1 (5.9)	0 (0.0)	0 (0.0)	1 (20.0)	0 (0.0)	0 (0.0)
Very	14 (82.4)	0 (0.0)	3 (100.0)	4 (80.0)	4 (80.0)	3 (100.0)
Missing	2	1	1	0	0	0
**Session 3**						
Not at all	1 (5.9)	1 (100.0)	0 (0.0)	0 (0.0)	0 (0.0)	0 (0.0)

A little	1 (5.9)	0 (0.0)	0 (0.0)	0 (0.0)	1 (20.0)	0 (0.0)
Somewhat	1 (5.9)	0 (0.0)	0 (0.0)	1 (20.0)	0 (0.0)	0 (0.0)
Very	14 (82.4)	0 (0.0)	3 (100.0)	4 (80.0)	4 (80.0)	3 (100.0)
Missing	2	1	1	0	0	0
**Session 4**						
Not at all	1 (5.9)	1 (100.0)	0 (0.0)	0 (0.0)	0 (0.0)	0 (0.0)
A little	1 (5.9)	0 (0.0)	0 (0.0)	0 (0.0)	1 (20.0)	0 (0.0)
Somewhat	1 (5.9)	0 (0.0)	0 (0.0)	1 (20.0)	0 (0.0)	0 (0.0)
Very	14 (82.4)	0 (0.0)	3 (100.0)	4 (80.0)	4 (80.0)	3 (100.0)
Missing	2	1	1	0	0	0
**Closing session**						
Not at all	1 (5.9)	1 (100.0)	0 (0.0)	0 (0.0)	0 (0.0)	0 (0.0)
A little	0 (0.0)	0 (0.0)	0 (0.0)	0 (0.0)	0 (0.0)	0 (0.0)
Somewhat	2 (11.8)	0 (0.0)	0 (0.0)	1 (20.0)	1 (20.0)	0 (0.0)
Very	14 (82.4)	0 (0.0)	3 (100.0)	4 (80.0)	4 (80.0)	3 (100.0)
Missing	2	1	1	0	0	0
**Phone/video sessions**						
Phone	12 (66.7)	1 (100.0)	3 (75.0)	2 (40.0)	4 (80.0)	2 (66.7)
Video	6 (33.3)	0 (0.0)	1 (25.0)	3 (60.0)	1 (20.0)	1 (33.3)
Missing	1	1	0	0	0	0
**Acceptability of phone/video sessions**						
Completely unacceptable	0 (0.0)	0 (0.0)	0 (0.0)	0 (0.0)	0 (0.0)	0 (0.0)
Unacceptable	0 (0.0)	0 (0.0)	0 (0.0)	0 (0.0)	0 (0.0)	0 (0.0)
No opinion	1 (5.6)	1 (100.0)	0 (0.0)	0 (0.0)	0 (0.0)	0 (0.0)
Acceptable	4 (22.2)	0 (0.0)	1 (25.0)	1 (20.0)	1 (20.0)	1 (33.3)
Completely acceptable	13 (72.2)	0 (0.0)	3 (75.0)	4 (80.0)	4 (80.0)	2 (66.7)
Missing	1	1	0	0	0	0

Note. Only data from participants who completed the acceptability questionnaires are included. Percentages were calculated excluding missing data.

Key: ACT= Acceptance and commitment therapy.

Table 3
Acceptability of ACT component, by site

Acceptability construct	Sites				
	Nottingham, n=2	Gateshead, n=4	King's Lynn, n=5	Woolwich, n=5	Whiston, n=3
**Overall acceptability score, n(%)**	12 (12-12)	15 (13-17)	13 (11-17)	17 (15-19)	15 (12-16)
**General Acceptability, n(%)**					
Completely unacceptable	0 (0.0)	0 (0.0)	1 (20.0)	1 (20.0)	0 (0.0)
Unacceptable	0 (0.0)	0 (0.0)	0 (0.0)	0 (0.0)	0 (0.0)
No opinion	1 (100.0)	0 (0.0)	0 (0.0)	0 (0.0)	0 (0.0)
Acceptable	0 (0.0)	1 (25.0)	2 (40.0)	0 (0.0)	0 (0.0)
Completely acceptable	0 (0.0)	3 (75.0)	2 (40.0)	4 (80.0)	3 (100.0)
Missing	1	0	0	0	0
**Affective attitude, n(%)**					
Strongly dislike	0 (0.0)	0 (0.0)	0 (0.0)	0 (0.0)	0 (0.0)
Dislike	0 (0.0)	0 (0.0)	0 (0.0)	0 (0.0)	0 (0.0)
No opinion	1 (100.0)	1 (25.0)	0 (0.0)	0 (0.0)	0 (0.0)
Like	0 (0.0)	0 (0.0)	3 (60.0)	1 (20.0)	1 (33.3)
Strongly like	0 (0.0)	3 (75.0)	2 (40.0)	4 (80.0)	2 (66.7)
Missing	1	0	0	0	0
**Burden, n(%)**					
No effort at all	0 (0.0)	0 (0.0)	1 (20.0)	0 (0.0)	0 (0.0)
A little effort	0 (0.0)	3 (75.0)	2 (40.0)	3 (60.0)	2 (66.7)
No opinion	1 (100.0)	0 (0.0)	0 (0.0)	0 (0.0)	0 (0.0)
A lot of effort	0 (0.0)	0 (0.0)	2 (40.0)	1 (20.0)	0 (0.0)
Huge effort	0 (0.0)	1 (25.0)	0 (0.0)	1 (20.0)	1 (33.3)
Missing	1	0	0	0	0
**Perceived effectiveness, n(%)**					
Strongly disagree	0 (0.0)	0 (0.0)	1 (20.0)	0 (0.0)	0 (0.0)
Disagree	0 (0.0)	0 (0.0)	0 (0.0)	0 (0.0)	1 (33.3)
No opinion	1 (100.0)	2 (50.0)	2 (40.0)	0 (0.0)	1 (33.3)
Agree	0 (0.0)	1 (25.0)	2 (40.0)	2 (40.0)	0 (0.0)
Strongly agree	0 (0.0)	1 (25.0)	0 (0.0)	3 (60.0)	1 (33.3)
Missing	1	0	0	0	0
**Coherence, n(%)**					
Strongly disagree	0 (0.0)	0 (0.0)	1 (20.0)	0 (0.0)	0 (0.0)
Disagree	0 (0.0)	1 (25.0)	1 (20.0)	0 (0.0)	1 (33.3)
No opinion	1 (100.0)	1 (25.0)	1 (20.0)	0 (0.0)	1 (33.3)
Agree	0 (0.0)	1 (25.0)	2 (40.0)	3 (60.0)	0 (0.0)
Strongly agree	0 (0.0)	1 (25.0)	0 (0.0)	2 (40.0)	1 (33.3)
Missing	1	0	0	0	0

Note. Only data from participants who completed the acceptability questionnaires are included. Percentages were calculated excluding missing data.
Key: ACT= Acceptance and commitment therapy.
