# Supplementary material for: Acceptability of Four Intervention Components Supporting Medication Adherence in Women with Breast Cancer: a Process Evaluation of a Fractional Factorial Pilot Optimization Trial
Source: Prev Sci. 2024 Jul 26;25(7):1065–78. doi: 10.1007/s11121-024-01711-9 (PMC11519312; doi:10.1007/s11121-024-01711-9)
Supplement: Supplementary file 5 — Online Resource 5 Illustrative quotes to supplement qualitative analysis (PDF 625 KB) [file 11121_2024_1711_MOESM5_ESM.pdf]

Supplementary material 4: Quotes to illustrate qualitative findings

Table 1. *Qualitative findings regarding the acceptability of the SMS intervention component*

	Key findings	Illustrative quote(s)
**Affective attitude**	• Most women liked the messages and the variety.	"I think it's a really good idea, yeah. Um I think the messages that are contained in them are quite helpful, useful and informative really and I think as opposed to, I, I envisaged that they would just be reminders where the extra information that they contained I thought was, um, was quite useful." *(≤50, C4[SMS, Web])*
	• A minority felt some messages were common sense, or out of place.	"Some I thought, well, well yeah that's really common sense…I think there was one about taking your medication on holiday and making sure you had it in your handbag…to me that's common sense." *(≥70, C4[SMS, Web])*
**Burden**	• Overall messages were low burden, and not intrusive.	"Uh, I thought the frequency was fine, cos it's only a message…a text message is is easy to either look at or ignore, isn't it?...So I, I, I didn't find it too intrusive." *(51-69, C3[SMS, ACT])*
	• Two participants felt daily messages were too frequent.	"I think when I was getting them like daily, there was points where I kind of would check my phone and go oh it's just that, because [laughs] I felt they were a bit too frequent." *(≤50, C3[SMS, ACT])*
	• Some participants may have opted out if not within trial setting.	"Had I not been within the trial, had, had this been kind of like real life [laughs] if that makes sense, um, but, I may well have done [opted out]… I figured that you know I was in the trial, and therefore I wanted to see what all the messages, because they were clearly different, how they changed and what the messages were like." *(51-69, C2[SMS, IL])*
**Coherence**	• Most women understood the messages were about building routines.	"They would be useful because at the start it's about, it's about getting, um, routines in place… and once you've got your routines in place, then things become easier and, and you're more likely to do that [take AET]." *(51-69, C2[SMS, IL])*
	• Some women felt the messages were a prompt.	"I guess to prompt, to prompt you to take them and suggest ways, an immediate way to suggest ways of how to help you to remind you to, to take them, um, and I suppose a way of sending you tips about things." *(≤50, C3[SMS, ACT])*
	• Some felt the messages emphasised the importance of taking AET.	"The reminder made me kind of talk to myself and say look you've got to take it, it's for your own good so just take it." *(≥70, C2[SMS, IL])*
	• One participant felt the messages were a form of social support.	"So my understanding of the trial, rightly or wrongly, was that it kind of just a sort of a friendly voice really, just to, sort of a gentle, a gentle support, a gentle we're still here for you and there is still somebody." *(51-69, C2[SMS, IL])*
**Perceived Effectiveness**	• Most women felt they had routines to take AET, but messages would be effective for those that didn't.	"I think if you, if you need a bit of help organising, I thought they would be very useful…they were just kind of coming from different angles… I can imagine that some women might just think ooh yeah that will work for me and try it, um because there was quite a few different ideas I think." *(51-69, C1[SMS, IL, ACT, Web])*
	• Some women felt personalising the timing of the messages would make them more helpful.	"Maybe if there had been one midday option, then you know that would've been nearer to the time that I take it... you know if you have like an 8am or a 6pm option." *(<50, C4[SMS, Web])*

Key: SMS=Short message service. 'C'= Condition, e.g. C1= Condition 1. Web= Website component. IL= information leaflet component. ACT= Acceptance and commitment therapy component. AET= Adjuvant endocrine therapy. ≤50= aged 50 or below. 51-69= aged 51 to 69. ≥70=aged 70 or above.

Article title: Acceptability of four intervention components supporting medication adherence in women with breast cancer: A process evaluation of a fractional factorial pilot optimization trial
Journal name: Prevention Science
Author names: Sophie M. C. Green, Nikki Rousseau, Louise H. Hall, David P. French, Christopher D. Graham, Kelly E. Lloyd, Michelle Collinson, Pei Loo Ow, Christopher Taylor, Daniel Howdon, Robbie Foy, Rebecca Walwyn, Jane Clark, Catherine Parbutt, Jo Waller, Jacqueline Buxton, Sally J. L. Moore, Galina Velikova, Amanda Farrin, Samuel G. Smith
Corresponding author: Sophie M. C. Green. Leeds institute of Health Sciences, University of Leeds. Email: s.m.c.green@leeds.ac.uk

Table 2. *Qualitative findings regarding the acceptability of the information leaflet intervention component*

	Key findings	Illustrative quote(s)
**Affective attitude**	• Several aspects of the leaflet were liked, including the quotes from other women, and information about side-effects.<br>• One participant felt they already knew the information, but liked having it written down.	"I think quotes from other women, I think that's, that is a good idea. I think that's something, you know, that you can relate to." *(51-69, C6[IL, Web])*<br>"The side effects table, I just thought that was just like really useful knowing that, that those things are side effects of the drugs that I'm taking. Um and I suppose it was just kind of reinforcing that what I was experiencing wasn't different from what other people experienced." *(51-69, C1[SMS, IL, ACT, Web])*<br>"I do think it's important to reinforce it and have it written down, so you've got that if you need to look back…for me I did a lot of research and reading around it anyway." *(51-69, C2[SMS, IL])*
**Burden**	• Concise and easy to read, without "medical jargon".	"It was easy to read, it wasn't written in complicated medical jargon so I understood what I was reading. And it wasn't a load of information bombarded at you. It was, it was concise, it was all I needed to know was in there." *(≥70, C2[SMS, IL])*
**Coherence**	• Most women understood the leaflet was aiming to provide information about AET.	"I feel like it, you were you were trying to support women to have, have the right information so they'd keep taking the tablets…I think the message was this is kind of how they work, this is what other people have to deal with, there are side effects but it's so important that you keep taking, taking them." *(51-69, C1[SMS, IL, ACT, Web])*
**Perceived Effectiveness**	• Some women reported being able to go back to the leaflet and re-read it to remind themselves of the benefits was helpful to remind them why they are taking AET.	"The usefulness of it for me is that I can always go back to it and read it over again to reassure myself that I'm doing the right thing." *(≥70, C2[SMS, IL])*<br>"There's a whole page with, you know what the benefits of taking it, and um it's very clear, you know, it can reduce it to come back and it can reduce your risk of dying from it. And if nothing else, it's that page that just makes me think, OK, I can cope with the side effects because I'm going to do everything possible to stop it coming back. I think that's, I think that's your message." *(51-69, C1[SMS, IL, ACT, Web])*

Key: SMS=Short message service. 'C'= Condition, e.g. C1= Condition 1. Web= Website component. IL= information leaflet component. ACT= Acceptance and commitment therapy component. AET= Adjuvant endocrine therapy. ≤50= aged 50 or below. 51-69= aged 51 to 69. ≥70=aged 70 or above.

Table 3. *Qualitative findings regarding the acceptability of the ACT intervention component*

ACT	Key findings	Illustrative quote(s)
**Affective attitude**	• Participants liked the practical, skills focus.	"Practical stuff, I think. So, you know, it wasn't kind of really like in depth kind of therapy, it was more about, OK, you know, these things are going on, let's think about positive ways that you can deal with some of this stuff… I thought that was really good." *(51-69, C1[SMS, IL, ACT, Web])*
	• A number of ACT skills were liked, with participants providing several explicit examples of how they have applied the skills to their lives.	"There was an audio where you had to imagine like a stream running past and like put your thoughts on a leaf and let them float past… I'm struggling with like menopausal symptoms, things like anxiety palpitations, hot flushes, and trying to kind of find ways to sort of breathe, take a moment out, just calm myself, like re-center…so that's an easy one for me now, that really stuck with me and I can like visualize and think about my thoughts." *(≤50, C3[SMS, ACT])*<br>"I was talking a lot about wanting to do something, but because I, I don't have the energy for work I, I wanted to do you know, something like volunteering where I only go if I feel up to it. And on Saturday I did a full days volunteering at, um, our local rugby club, you know and Oh my God like it just it's like a new lease of life to be able to go out and spend the day out. My confidence was up to talk to people, you know." *(<50, C7[ACT, Web])*
	• Support sessions from the therapist were liked by all participants overall.	"She was lovely, um, and, you know I got, I felt as though we got on well together and we, you know, we could chat, or I could chat easily to her…I didn't feel as though I couldn't say anything, or I was guarded at all, I was, you know, quite comfortable talking to her." *(51-69, C3[SMS, ACT])*
	• Most participants felt the timing of the sessions were good, as other support had ceased. One participant felt they were not ready for the sessions.	"After I'd finished the radio therapy there was nothing for a long time and that felt, that felt hard to cope; how do I know the cancer has gone…I mean yes I have phone calls to phone people but it was just that I should be feeling happy about the fact that it's all over, but having the sessions with the psychologist helped me to cope with that, and get over it, and not feel as dependent on needing to go to the hospital all the time." *(51-69, C5[IL, ACT])*<br>"Just the opportunity to talk was the most helpful thing. Um, and I could see where it could go with like the, the mindfulness and acceptance that way, I could, I could see where it could go, um, I felt for me it was all too raw still." *(51-69, C7[ACT, Web])*
	• One participant felt some pressure to talk in the sessions to fill the time.	"She listened really well, but I didn't feel like I got too much from her back. I felt like I was the one that had to do, so I felt a little bit sort of anxious that I had to keep talking, if you know what I mean." *(51-69, C3[SMS, ACT])*
**Burden**	• Liked the online delivery and flexibility of sessions- made it easier to attend.	"If I hadn't of been, if it hadn't of been virtual, I probably would have missed three sessions…because I wasn't well enough to do, to get out." *(≤50, C7[ACT, Web])*
	• Weekly sessions too close together- need more time to practice skills.	"I felt like I didn't have much time to sort of do the booklet and work on it before I was having a review...Two to three weeks [between sessions] I would have, I would have felt better. I would have felt like I'd worked on it more and probably could have asked more questions." *(51-69, C3[SMS, ACT])*

	• Therapy was physically and emotionally challenging- having sessions in the morning and then going back to work was difficult.	"The second I start talking about how I feel and my emotions I get very tearful, and, um, I then sort of have to talk and think about things that I, I really suppress a lot of the time, and then I get this physical reaction to it with the tears, um, and so I would get the, the end of the session and I feel like I've been steam-rollered basically, um, and, and it takes me a while to, sort of, get my equilibrium back. Um, and I think the, the, challenge for me was the timing of the sessions, often the only time slot I could have, um, would be in the mornings and then having to then put myself back together and go to work was quite difficult." *(51-69, C7[ACT, Web])*
**Coherence**	• Overall understanding that ACT was teaching skills and coping mechanisms.	"It's giving you those skills to cope because there's, there's such a lot going on, and I think until you go, like I didn't even realise myself how, how much I'd still be dealing with now, um so I suppose it kind of arms you with the skills for what else might come along, you know, come your way while you're going along this journey if you like." *(≤50, C3[SMS, ACT])*
	• Some participants were initially unsure about how ACT would help them.	"Well, in the beginning, it was, the first two sessions, it was kind of, um, hard to get my head around it. Do you know, I didn't really see a plan for it or what it was doing. But then by the third one, I felt that it was doing very, you know, I could understand it more, and I was figuring it out, um, I found it very good from then on in." *(≤50, C7[ACT, Web])*
**Perceived Effectiveness**	Multiple experiences were shared regarding perceived effectiveness:<br>• How ACT had helped take AET<br>• Reduced psychological distress<br>• Helpful to return to work<br>• Helped to cope with side-effects of AET	• "I was gonna pack it in [taking AET] and, um this [ACT] sorta gave me the positivity um, so that when I did speak to my, um, doctor when she rang the the cancer doctor, the oncologist, um, I was going to ask her to take me off it, but I decided to give it another chance and that happened about the same time as starting this." *(51-69, C7[ACT, Web])*<br>• "I found them very, very therapeutic, especially mentally. I think that that's helped my mental health 100%. You know, it really has, it really has helped that side of it." *(51-69, C7[ACT, Web])*<br>• "I think as well going through those, some of those strategies and thinking, you know, make you think what's important and what's not, with going back to work, it sort of helped to know, I was a bit like well I'm not gonna stress about this anymore." *(51-69, C3[SMS, ACT])*<br>• "I got such a lot out of it, um, I was able to relax more, my sleeping came back… I sorted my hot flushes without taking drugs." *(51-69, C5[IL, ACT])*

Key: SMS=Short message service. 'C'= Condition, e.g. C1= Condition 1. Web= Website component. IL= information leaflet component. ACT= Acceptance and commitment therapy component. AET= Adjuvant endocrine therapy. ≤50= aged 50 or below. 51-69= aged 51 to 69. ≥70=aged 70 or above.

Table 4. *Qualitative findings regarding the acceptability of the website intervention component*

	Key findings	Illustrative quote(s)
**Affective attitude**	• Some women felt it was beneficial to see videos of other women.<br>• However, one participant felt the videos were too stereotypical.<br>• One participant felt the website was not aesthetically pleasing.<br>• A few participants found the information too general and vague in places.<br>• Some women liked the honesty of the evidence ratings for the side-effect management strategies, but others did not feel this was helpful.	"It's actually so beneficial when you're watching other people who have gone through it, um, when you're just starting, it really, really helps." *(51-69, C7[ACT, Web])*<br>"More relatable age, more relatable people to me as well, you know I'm not typical grey haired 50-60 year old, that's awful but you know what I mean." *(≤50, C4[SMS, Web])*<br>"It seemed quite, not overly user friendly and a little bit clunky, I don't think it's attractive aesthetically…It's all very boxy…not soft, or not approachable really." *(≤50, C4[SMS, Web])*<br>"I accessed it because I was experiencing joint pain, bone pain...the information about bone pain was that vague that I came away thinking well it didn't really answer my question you know what I mean, it, it, it's informative but not enough. It's too vague." *(≤50, C4[SMS, Web])*<br>"I mean I liked the fact that it gave the evidence. You know, I'm. I kind of like working on sort of facts. It kind of work, it worked for me. It wasn't a great revelation, but it was like, you know, some of these things are worth trying, they might not help. But you know, there's no particular evidence, but you never know." *(51-69, C1[SMS, IL, ACT, Web])*<br>"I mean it's almost saying right there's the suggestions however there's no evidence. It's just like taking and giving on one hand and then saying well there's no evidence for this...if there's no evidence to it it's almost like well it shouldn't even be there then." *(≤50, C4[SMS, Web])*
**Burden**	• The website modality was acceptable.	"I liked it being online, I think you know for, um, certainly people, most people these days, um, you know, are familiar with accessing websites and navigating around them now." *(51-69, C6[IL, Web])*
**Coherence**	• Participants generally understood the website was to provide side-effect self-management strategies.	"They can dig into things that would help them manage side effects, that's useful." *(51-69, C6[IL, Web])*
**Perceived Effectiveness**	• Some women acknowledged the website would be helpful for those who haven't researched coping strategies.<br>• Some women felt the website didn't teach them anything new.	"It was all things that made sense, um, were logical and you know, I can imagine that if you weren't the sort of person that would've thought of all those things already, it would've been very helpful." *(51-69, C7[ACT, Web])*<br>"Nothing was really surprising. And nothing's really light bulb moment… It, it was an interesting read, but I don't think it massively changed things for me." *(51-69, C1[SMS, IL, ACT, Web])*

Key: SMS=Short message service. 'C'= Condition, e.g. C1= Condition 1. Web= Website component. IL= information leaflet component. ACT= Acceptance and commitment therapy component. AET= Adjuvant endocrine therapy. ≤50= aged 50 or below. 51-69= aged 51 to 69. ≥70=aged 70 or above.
