# Supplementary material for: Acceptability of Four Intervention Components Supporting Medication Adherence in Women with Breast Cancer: a Process Evaluation of a Fractional Factorial Pilot Optimization Trial
Source: Prev Sci. 2024 Jul 26;25(7):1065–78. doi: 10.1007/s11121-024-01711-9 (PMC11519312; doi:10.1007/s11121-024-01711-9)
Supplement: Supplementary file 7 — Online Resource 7 Intervention component adaptations (DOCX 22 KB) [file 11121_2024_1711_MOESM7_ESM.docx]

**Online resource 7- Intervention Component Adaptations**

| **Table 1**  *Summary of key adaptations for each intervention component.* | | | |
| --- | --- | --- | --- |
| **Component** | **Suggestion or feedback from participant** | **Action taken (Y/N)** | **Detail of action taken/ reason why action not taken** |
| SMS | Option to select what time of day messages could be sent | Y | Participants can select messages to be sent morning, lunchtime or evening |
| SMS | Some messages felt out of place/ too obvious | Y | Messages reviewed with PPI group |
| SMS | Many women already had routines in place to take their medication | N | Women with all levels of adherence can be recruited as the intervention components could prevent decline of medication adherence, as well as improve low adherence. |
| IL + Web | Some participants did not recall being sent these components; potentially mixed up in large email with other trial documents. | Y | Send leaflet and website components 1 week after randomization, separate to other trial documents. |
| Website | Videos with younger, more relatable women, | N | The current videos are from women from a range of ages. Our videos were sourced from health talk. There are no additional videos available that we can use. We do not have the resources to create our own videos. |
| Website | Make website more aesthetically pleasing. | N | We do not have the resources to change the overall aesthetic of website. |
| Website | Add more specific information e.g., about when to see a doctor after joint pain. | N | The website already advises that women should see a doctor if their pain is severe. |
| Website | Mixed opinions about evidence ratings for side-effect self-management strategies. | Y | “Evidence” changed to “scientific evidence” to make it clearer that we are only referring to scientific evidence. Wording amended based on advice from our PPI group. We have also made it clearer that lifestyle changes such as exercise can be beneficial for overall health. |
| ACT | Weekly sessions were too close together- not enough time to complete home practice. | Y | There is now more flexibility in the timing of the sessions; sessions should be completed within 3 months from the first session and can be delivered weekly or fortnightly within this. |
| ACT | Apprehension at the beginning of sessions with little understanding of what to expect and how the sessions may be of benefit. | Y | More information added to the patient information sheet about what to expect from the ACT sessions, and what the sessions will involve. |
| ACT | Felt pressure to keep talking in the sessions. | Y | Inform therapists that they can shorten the sessions if the full time is not needed. Additional element included in therapist training regarding how to approach sessions if the participant is struggling to engage in self-reflection. |
| SMS: Short message service. IL: information leaflet. ACT: acceptance and commitment therapy. Web: Website. PPI: Patient and public involvement. AET: adjuvant endocrine therapy. | | | |
