# Supplementary material for: Acceptability of Four Intervention Components Supporting Medication Adherence in Women with Breast Cancer: a Process Evaluation of a Fractional Factorial Pilot Optimization Trial
Source: Prev Sci. 2024 Jul 26;25(7):1065–78. doi: 10.1007/s11121-024-01711-9 (PMC11519312; doi:10.1007/s11121-024-01711-9)
Supplement: Supplementary file 1 — Online Resource 1 ROSETA conceptual model (PDF 166 KB) [file 11121_2024_1711_MOESM1_ESM.pdf]

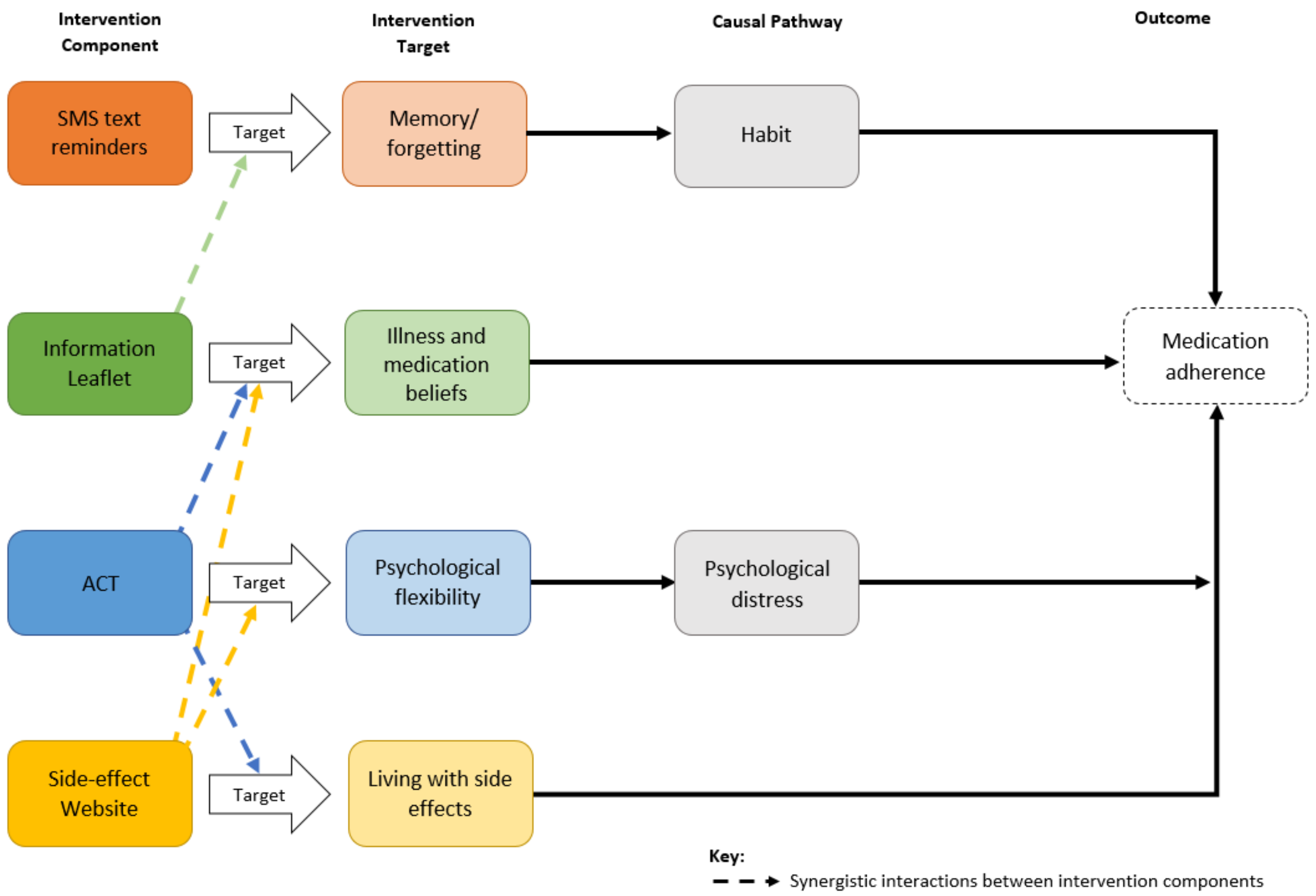

Figure 1

ROSETA conceptual model. Taken directly from Green et al., (2022).

Green, S. M. C., French, D. P., Graham, C. D., Hall, L. H., Rousseau, N., Foy, R., Clark, J., Parbutt, C., Raine, E., Gardner, B., Velikova, G., Moore, S. J. L., Buxton, J., Collinson, M., Ellison, R., Wilkes, H., Hartley, S., Mason, E., Farrin, A., . . . Smith, S. G. (2022). Supporting adjuvant endocrine therapy adherence in women with breast cancer: the development of a complex behavioural intervention using Intervention Mapping guided by the Multiphase Optimisation Strategy. *BMC Health Services Research*, *22*(1), 1081. https://doi.org/10.1186/s12913-022-08243-4
